# Endothelial-to-Mesenchymal Transition, Vascular Inflammation, and Atherosclerosis

**DOI:** 10.3389/fcvm.2020.00053

**Published:** 2020-05-05

**Authors:** Pei-Yu Chen, Martin A. Schwartz, Michael Simons

**Affiliations:** ^1^Department of Internal Medicine, Yale Cardiovascular Research Center, New Haven, CT, United States; ^2^Department of Cell Biology, Yale University School of Medicine, New Haven, CT, United States

**Keywords:** endothelial-to-mesenchymal transition (EndMT), inflammation, TGFβ (transforming growth factor β), FGF (fibroblast growth factor), endothelium, atherosclerotic plaques

## Abstract

Atherosclerosis is a chronic progressive disease characterized by vascular inflammation and growth of atherosclerotic plaque that eventually lead to compromise of blood flow. The disease has proven to be remarkably resistant to multiple attempts at meaningful reversal including recent strategies targeting selective inflammatory mediators. Endothelial-to-mesenchymal transition (EndMT) has emerged as a key driver of both vascular inflammation and plaque growth. A deeper understanding of EndMT provides new insights into the underlying biology of atherosclerosis, suggests likely molecular mechanism of atherosclerotic resistance, and identifies potential new therapeutic targets.

## Inflammation and Atherosclerosis

Atherosclerosis is a complex, slowly developing disease characterized by a gradual transformation of intimal fatty streaks into full-blown plaques composed of activated endothelial and smooth muscle cells, macrophages, lymphocytes, and large amounts of extracellular matrix. The process is initiated by vascular injury–induced primarily by hyperlipidemia, albeit other factors, such as tobacco, hypercysteinemia, diabetes, and hypertension, also play a role (1, 2). Atherosclerosis occurs preferentially in areas where fluid shear stress is low and shows complex changes in direction during the cardiac cycle; these flow patterns are often grouped together under the term “disturbed shear stress” (DSS), which we use hereafter (3). The hallmark of an atherosclerotic vessel is chronic vascular wall inflammation; indeed, the entire syndrome can be thought of as unresolved vascular inflammatory response (4).

Despite seemingly clear understanding of its pathogenesis, the diseases proved remarkably difficult to control and, especially, reverse. While aggressive lipid lowering slows down plaque growth and stabilizes, to an extent, vulnerable lesions, no meaningful regression occurs, and vascular inflammation is largely unaffected. This led to attempts to directly control vascular inflammation by targeting specific actors, such as interleukin 1β (IL-1β), or using general inflammation inhibitors, such as methotrexate, without clear success (5).

Recent evidence has pointed to endothelial-to-mesenchymal transition (EndMT) as a key process in vascular inflammation in atherosclerosis (6, 7). Intriguingly, EndMT appears also to be involved in other cardiovascular conditions including pulmonary hypertension, renal dysfunction, and vascular malformations, suggesting a common pathological basis for multiple vascular diseases (8, 9). EndMT has also been observed in the aging vasculature and may contribute to the aging process itself (10). This review will focus on EndMT, its biological basis, and its role in atherosclerosis.

## Inflammation, Shear Stress, and EndMT

EndMT is a recently described biological process in which endothelial cells lose their characteristic cobblestone appearance and acquire the elongated shape typical of mesenchymal cells, gaining increased migratory, and proliferative capacity but diminishing barrier function (8, 9). Initially described during development of cardiac atrioventricular valves, EndMT has now been observed in various pathologic conditions characterized by abnormal shear stress, vascular injury, and chronic inflammation. At the molecular level, endothelial marker genes, such as vascular endothelial growth factor receptor 2 (VEGFR2), VE-cadherin (Cdh5), and endothelial nitric oxide synthase (NOS3), are reduced, and “mesenchymal” genes including fibroblast specific protein 1 (FSP1), fibronectin (FN1), and N-cadherin (Cdh2) are increased (11). Whether this constitutes a true transdifferentiation or molecular mimicry is the subject of intense debates in the literature. Importantly, thus transformed, “EndMT'd” endothelial cells become intensely proinflammatory expressing high levels of leukocyte adhesion molecules (intercellular adhesion molecule 1, vascular cell adhesion molecule 1) and various cytokines and growth factors (12). While EndMT *per se* is clearly a pathologic response, it is probably best viewed as the most extreme phenomenon in the spectrum of endothelial activation. Indeed, any endothelial cell activation includes some EndMT features including expression of “mesenchymal” genes. If this activation is persistent, it may progress, over time, to endothelial dysfunction and eventually to a full-blown cell fate change (EndMT) (12).

Transforming growth factor receptor β (TGFβ) signaling is central to EndMT, albeit Wnt/β-catenin and Notch signaling may also contribute in certain settings. The common theme seems to be increased expression of transcription factors Snail, Slug, Twist, LEF-1, ZEB1, and ZEB2 that repress expression of endothelial and/or activate expression of mesenchymal genes (13). TGFβ family consists of three closely related proteins (TGFβ1, TGFβ2, and TGFβ3), with TGFβ1 being the most abundant isoform in most tissues. TGFβ signaling is highly pleiotropic, playing crucial roles in embryogenesis, cell differentiation, immune system development, inflammation, and wound repair (14–16). The signaling is tightly controlled at multiple levels including ligand expression, activation, and receptor expression. TGFβs are secreted in a biologically inactive (latent) form. Once free from its latency-associated peptide dimer, TGFs can bind a low-affinity cell surface receptor β-glycan (TGFβR3) followed by binding to two high-affinity serine/threonine kinase receptors (TGFβR1 and TGFβR2) (17). TGFβ binding to the constitutively active TGFβRII leads to formation of a tetrameric complex (TGFβRII dimer and two TGFβR1s). This results in TGFβR1 activation and initiation of downstream signaling. The canonical signaling pathway is mediated by TGFβR1 phosphorylation of Smad2 and Smad3 that induces their heterodimerization with Smad4 (17). Thus activated, Smad complexes then translocate to the nucleus and, in cooperation with other transcription factors, regulate expression of a large number of target genes. Noncanonical signaling involves activation of MAPK and Rho family GTPases pathways (18).

**Figure 1:**
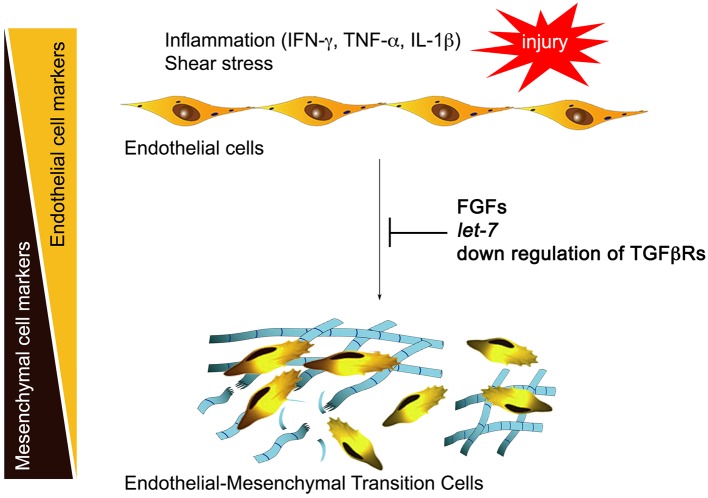
Inflammation and endothelial-to-mesenchymal transition (EndMT). Inflammatory mediators including interferon γ (IFN-γ), tumor necrosis factor α (TNF-α), and interleukin 1β (IL-1β) induce downregulation of endothelial fibroblast growth factor (FGF) receptors, reducing FGF signaling input. This leads to a large fall in let-7 miRNA levels and activation of transforming growth factor receptor β (TGFβ) signaling, initiating EndMT. Restoration of FGF signaling, endothelial let-7 miRNA levels, or suppression of endothelial TGFβ receptor expression arrest EndMT development.

Normal adult quiescent endothelial cells have a very low expression of TGFβR1, rendering these cells nearly completely resistant to TGFβ stimulation and thus EndMT (6). This is controlled by continuous fibroblast growth factor (FGF) signaling that maintains high expression of let-7 family of microRNAs (miRs). A decline in FGF signaling leads to a dramatic (50- to 100-fold) decrease in let-7 miRs levels and a rapid increase in TGFβR1, thereby upregulating TGFβ signaling. In contrast, continued FGF signaling input, high endothelial let-7 levels, and suppression of TGFβRs expression maintain endothelial normalcy (Figure 1). This reciprocal relationship between TGFβ and FGF signaling outputs becomes important in atherosclerosis because vascular inflammation effectively suppresses FGF signaling by profoundly reducing expression of FGF receptor 1 (FGFR1, the principal endothelial FGF receptor), thereby increasing TGFβR1 expression and initiating EndMT (6).

## Fluid Shear Stress

Shear stress from blood flow is a major determinant of vascular morphogenesis and remodeling, as well as initiation and progression of atherosclerosis (19–21). Endothelial responses to shear play important roles both in normalcy and disease. Regions of arteries that branch or curve sharply exhibit irregular flow patterns with lower magnitude of shear stress and complex changes in direction during the cardiac cycle, termed DSS. DSS induces modest but chronic activation of inflammatory pathways in the endothelium and sensitizes it to other inflammatory mediators, greatly amplifying responses. On the other hand, endothelial cells under higher, and unidirectional (physiological) shear stress suppress inflammatory pathways and downregulate responses to inflammatory cytokines (21, 22). As expected from the key role of inflammation in sensitizing the endothelium to TGFβ, DSS is sufficient to induce EndMT *in vitro* (where TGFβ is abundantly present) and *in vivo*. (6, 23, 24) The latter is in part due to a decrease in FGFR1 expression in DSS regions and results in a reduction in protective FGF and activation of pathogenic TGFβ signaling (6, 19). Conversely, physiological shear stress limits TGFβ signaling with a major role for the atheroprotective, anti-inflammatory Erk5-KLF2 pathway (25–27). Elevated TGFβ signaling in response to DSS is thus poised to contribute to EndMT and selective atherogenesis in these regions.

## EndMT and Atherosclerosis

Several recent studies have shown the link between EndMT and atherosclerosis. In atherosclerotic human coronary arteries, a high proportion of luminal endothelial cells covering the plaques expresses smooth muscle cell (SMC) and mesenchymal markers (6). Critically, the extent of EndMT strongly correlates with the extent of atherosclerosis and inversely correlates with expression of FGFR1 (6). Similarly, ApoE null mice on high-fat/high-cholesterol diet mice show a progressive increase in EndMT as the extent of atherosclerosis increases. Interestingly, atherosclerosis-prone sites (areas of DSS) demonstrated lower level of FGFR1 staining compared to atherosclerosis-resistant regions of the arterial vasculature, suggesting that DSS downregulates FGFR1 expression (6).

The link between FGFR1 expression and atherosclerosis was further demonstrated in ApoE null mice with endothelial-specific deletion of the FGF receptors signaling scaffold protein fibroblast growth factor receptor substrate 2α (FRS2α), which fully disrupts FGF signaling. On a high-fat/high-cholesterol diet, mice with endothelial FRS2α deletion developed much more extensive atherosclerotic plaques with larger necrotic cores. Furthermore, there was a complete loss of high shear stress protection leading to plaque formation in normally atherosclerosis-resistant areas, thus further linking anti-atherosclerotic effects of high shear and FGF signaling with EndMT and atherosclerosis (6).

Inflammatory cytokines also regulate FGFR1 expression: exposure of primary ECs *in vitro* to IFN-γ, TNF-α, and IL-1β leads to reduced FGFR1 expression. Importantly, while relatively high doses were required for each individual cytokine to inhibit FGFR1 expression, a combination of two or more profoundly suppressed FGFR1 expression at much lower doses (6). These findings suggest that inhibition of any one inflammatory cytokine is unlikely to be effective in treatment of atherosclerosis.

Subsequent studies examining the contribution of EndMT-derived fibroblast- and myofibroblast-like cells in atherosclerotic lesions confirmed high frequency of EndMT in plaques with up to 46% of fate-mapped ECs expressing fibroblast marker after 30 weeks of high-fat/high-cholesterol diet. (7, 27) Of note, analysis of gene expression data analysis revealed EndMT cell gene expression pattern is different from authentic endothelial cells and fibroblasts, suggesting that the observed phenotype “switch” is not a true transformation.

**Figure 2:**
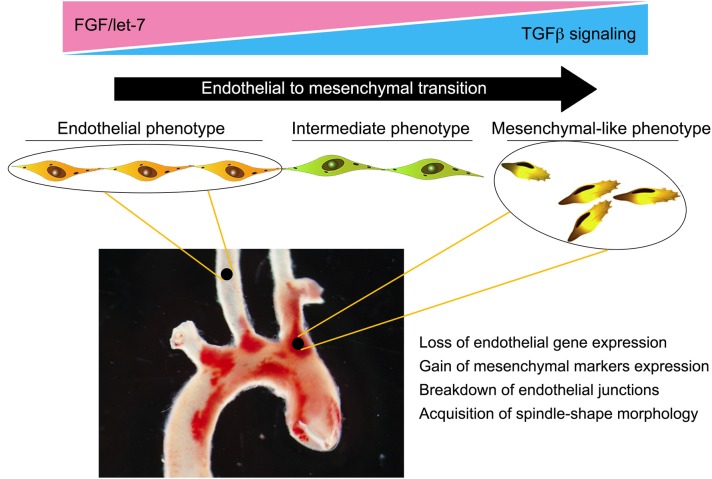
Endothelial-to-mesenchymal transition (EndMT) in atherosclerosis. Endothelial cells in atherosclerotic vessels can, as a result of chronic vessel wall inflammation, undergo EndMT. This is a gradual process, with the majority of endothelial cells progressing to an intermediate phenotype characterized by a partial loss of endothelial-specific gene expression and acquisition of “mesenchymal” features. However, a significant number can progress to a fully blown mesenchymal phenotype characterized by a near-complete loss of endothelial fate gene expression and acquisition of mesenchymal fate gene expression. This results in breakdown of endothelial cell junctions, increased vascular leakiness, and promotion of inflammation, thereby establishing a feed forward loop and driving atherosclerosis progression.

Taken together, these data highlight the importance of EndMT in atherosclerosis and trace its development to the loss of protective FGF signaling due to abnormal low shear and vascular wall inflammation (Figure 2). Yet while strongly suggesting a pathogenic role for EndMT in atherosclerosis, these studies did not establish a causal relationship. To test the effect of silencing endothelial TGFβ signaling on atherosclerosis, Chen et al. (28) created endothelial fate-mapped mice carrying floxed TGFβR1 and TGFβR2 alleles under control of an inducible Cdh5 promoter on an ApoE^−/−^ background. Induced deletion of TGFβR1 and TGFβR2 genes in adult mice at the time of initiation of high-fat/high-cholesterol diet resulted in ~60% reduction in the size of atherosclerotic plaques. Importantly, the frequency of EndMT was dramatically reduced, as was expression of endothelial leukocyte adhesion molecules and vessel wall inflammation (28). To test if inhibition of EndMT would lead to regression of fully established lesions, endothelial TGFβR1/R2 deletion was induced in mice with fully developed plaques with the animals either continued on the high-fat/high-cholesterol diet or switched to the normal chow diet. In both cases, inhibition of endothelial TGFβ signaling induced a profound (70% over 2 months) regression of the plaque and resolution of vascular inflammation. Single-cell RNA-seq analysis of endothelium in the ApoE^−/−^ mice demonstrated the presence of a population of endothelial-derived cells characterized by low expression of endothelial and high expression of mesenchymal markers and a dramatic increase in expression of genes associated with inflammation. Following endothelial deletion of TGFβR1/R2, this population was markedly decreased, in keeping with reduced atherosclerosis and small plaque size (28).

The suggestion arising from these data that endothelial TGFβ signaling is proinflammatory seemingly contradicts the consensus that this signaling pathway is anti-inflammatory (17, 29–31). This problem was directly addressed by an experiment that examined the genetic signature of TGFβ in endothelial, smooth muscle, macrophages, and T cells. There was a distinct TGFβ-induced gene expression profile in every cell type. Strikingly, endothelial TGFβ stimulation induced expression of numerous cytokines and cytokine receptors, as well as various leukocyte adhesion molecules, findings consistent with proinflammatory effects of stimulation. When tested further *in vivo*, endothelial deletion of TGFβ receptors resulted in a profound reduction of inflammatory cell accumulation at the site of TNF-α injection (28).

## Therapeutic Approach to Atherosclerosis: A Reappraisal

These data establish that EndMT is a key contributor to the development and, importantly, progression of atherosclerosis. Unlike transient inflammation, sustained inflammation due to hyperlipidemia and DSS that does not resolve leads to EndMT due to inflammation-driven suppression of protective endothelial FGF signaling. Importantly, this establishes a positive feedback loop: EndMT begets inflammation, which begets more EndMT (12). Now even if the initiating insult (hypercholesterolemia) is removed, the process will continue. This likely explains why even a profound suppression of cholesterol levels only slows down but does not reverse atherosclerosis in patients. Suppression of a single cytokine is also predicted to have little benefit, as recently demonstrated in the CANTOS trial that tested the inhibitory anti-IL-1β antibody canakinumab. Despite some positive trends, overall results were less than robust, and side effects included a significantly higher incidence of fatal infection and sepsis (5). If disease regression is the goal, then addressing the root cause of this resistance becomes critical to success.

A broader approach to suppressing vascular inflammation using low-dose methotrexate also failed to show any benefit in the CIRT trial (32). Low-dose colchicine showed some survival benefit in patients with recent myocardial infarction in COLCOT trial, but whether this is due to antiplaque effects has not been examined (33). Finally, it should be noted that while TGFβ has been identified as a central driver of EndMT, systemic targeting of TGFβ pathway, using either anti-TGFβ or TGFβR antagonists, is not practical given a very complex nature of TGF biology (15, 17, 30). Indeed, systemic as well as T-cells or smooth muscle-specific inhibition of TGFβ signaling has been associated with activation of T cells (29), loss of protection against vascular inflammation (31), and accelerated progression of atherosclerosis (34).

An alternative to systemic approach to inhibition of TGFβ signaling is the development of endothelial-targeted therapies. Recent advances in nanomedicine led to discovery of several classes of nanoparticles capable of targeting different organs (35). One such therapy specifically targeting the liver was recently approved by the US Food and Drug Administration (36). Another class of nanoparticles, designated as 7C1, targets the endothelium of large- and medium-size vessels (37, 38). Several recent studies showed biological efficacy of 7C1-mediated gene suppression or gene delivery in mice and nonhuman primates (39–43). Importantly, when used to deliver TGFβR1/R2 RNAi to atherosclerotic vessels in ApoE^−/−^ mice, 7C1 nanoparticles were effective in suppressing EndMT and reversing atherosclerotic plaque (28). The advantages of this approach include enhanced selectivity of TGFβ signaling suppression and the ability to deliver higher doses than would be possible with systemic therapy. Whether this approach is translatable to larger animal models and ultimately to patients requires further studies.

## Conclusions

While the importance of vascular inflammation in atherosclerosis has long been recognized, the factors responsible for its resistance to therapies and continued progression remained unknown. Further, treatments based on lipid lowering or single-cytokine inhibition at best slow but do not arrest or reverse disease. The emergence of EndMT as the central mechanism controlling ongoing vessel wall inflammation now promises to open new therapeutic. EndMT is driven by high TGFβ signaling that is surprising and perhaps uniquely proinflammatory in the endothelium. Effective endothelial-specific suppression of this signaling cascade in an endothelial-specific manner appears not to suppress vessel wall inflammation and arrest atherosclerotic plaque growth but also to induce substantial regression of mature atherosclerotic lesions in mouse models.

## Author Contributions

All authors participated in writing and editing of the manuscript.

## Conflict of Interest

MS and P-YC are holders of provisional US Patent Applications 62/311,086 and 62/406,732 dealing with endothelial-specific treatment of atherosclerosis. MS and P-YC are scientific founders of VasoRx, Inc. MS is the chair of VasoRx, Inc. Scientific Advisory Board. The remaining author declares that the research was conducted in the absence of any commercial or financial relationships that could be construed as a potential conflict of interest.
